# Society of Veterinary Graduates of Wisconsin

**Published:** 1891-04

**Authors:** 


*Society of Veterinary Graduates of Wisconsin.*—The Society of Veterinary Graduates of Wisconsin was organized at Madison, March 18, 1891. The following officers were elected: President, V. L. Atkinson, V S., Milwaukee; Vice-President, J. L. Scott, V. S., Beaver Dam; Secretary, W. P. Freeman, D. V. S., New Richmond; Treasurer, C. H. Ormond, D. V. S., Milwaukee.

Censors: J. F. Raub, D. V. S., Monroe; L. R. Baker, V. S., Madison; A. Kurtz, V. S., Appleton.

A Constitution and By-Laws were adopted, which limit the members to graduates of such colleges as are declared to be regular Veterinary Colleges by the Society. An important feature provides for blanks to be issued a month prior to every meeting with notice of business matters, on which the members may vote if they cannot be present.

